# A Rare Case of Pancreatic Tuberculosis Diagnosed via Endoscopic Ultrasound-Guided Fine Needle Aspiration and Polymerase Chain Reaction

**DOI:** 10.7759/cureus.8795

**Published:** 2020-06-24

**Authors:** Gilles J Hoilat, Manasik Abdu, Judie Hoilat, Lorenzo Gitto, Abdul Q Bhutta

**Affiliations:** 1 Internal Medicine, State University of New York (SUNY) Upstate Medical University, Syracuse, USA; 2 Internal Medicine, Alfaisal University, Riyadh, SAU; 3 Internal Medicine, Loyola University Medical Center, Chicago, USA; 4 Pathology, State University of New York (SUNY) Upstate Medical University, Syracuse, USA; 5 Gastroenterology, State University of New York (SUNY) Upstate Medical University, Syracuse, USA

**Keywords:** endoscopic ultrasound (eus), endoscopy ercp, pancreatic malignancy

## Abstract

Pancreatic tuberculosis (TB) is a very rare condition even in endemic areas of the world where the disease is considered to be highly prevalent. The presenting features are usually vague and its radiological features mimic pancreatitis and pancreatic malignancy. We present a case of a 26-year-old active military male, originally from Virginia with no past medical history who presented to the ED with a two-week history of abdominal pain, increased nausea and vomiting, decreased appetite, increased darkening of his urine, and pale-colored stools. His physical examination was remarkable for conjunctival icterus as well as generalized abdominal tenderness. His laboratory results were remarkable for a total bilirubin of 4.7 mg/dL, direct bilirubin of 3.9 mg/dL, and alkaline phosphatase of 583 U/L. A CT scan was performed showing an intrahepatic dilatation and abrupt obstruction of the common bile duct at the level of a mass. Subsequent MRI of the abdomen was performed which showed a pancreatic mass at the uncinate process obstructing the common bile duct and causing intrahepatic bile dilation. The patient was deemed a surgical candidate and endoscopic retrograde cholangiopancreatography (ERCP)/endoscopic ultrasound (EUS) was performed for the sake of staging and showed a biliary compression in the middle of the common bile duct for which a stent was placed, and fine-needle aspiration (FNA) of the pancreatic mass was performed which was consistent with necrotizing granulomatous lymphadenitis. After further diagnostic studies, the patient was diagnosed with pancreatic TB. This case highlights the unusual presentation of extrapulmonary TB as well as the importance of EUS-guided FNA in diagnosing pancreatic TB which was presumed to be a malignant mass and candidate for unnecessary surgical resection.

## Introduction

Pancreatic tuberculosis (TB) is a rare type of abdominal TB that usually presents as a pancreatic tumor mimicking carcinoma [1]. There are no specific clinical or radiological findings pertaining to pancreatic TB. The incidence of pancreatic TB is rare even in patients with miliary TB. In a review of 300 Indian patients with abdominal TB, there was not a single case reporting the involvement of the pancreas [2]. Auerbach and Paraf et al. have examined the autopsy cases of miliary TB and reported that the incidence of pancreatic TB is 4.7% (of 297) and 2.1% (of 526) respectively [3-4]. However, within the past decade, there has been a rise in the number of cases indicating pancreatic involvement by TB, possibly due to the advancement of technology and the availability of better diagnostic modalities [1]. We present a case of pancreatic TB masquerading as pancreatic adenocarcinoma and successfully diagnosed with Mycobacterium polymerase chain reaction (PCR). 

## Case presentation

**Figure 1:**
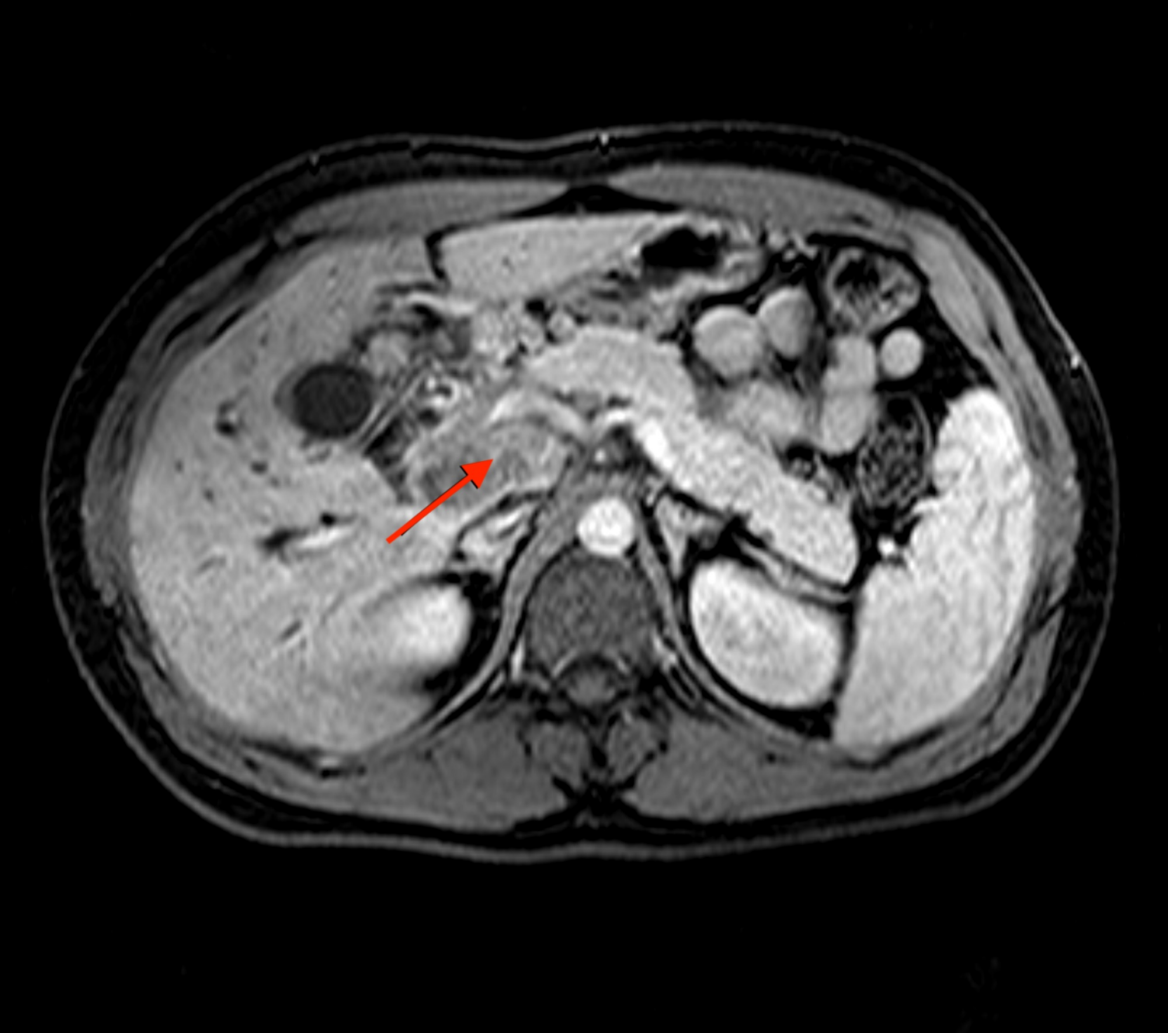
Abdominal MRI with gadolinium injection depicting an irregularly enhancing lobulated mass at the uncinate process of the pancreas measuring 2 cm x 1.9 cm.

A 26-year-old active military male, originally from Virginia with no history of TB and no past medical history presented to the ED with a two-week history of abdominal pain, increased nausea and vomiting, decreased appetite, increased darkening of his urine, and pale-colored stools. His physical examination was remarkable for conjunctival icterus as well as generalized abdominal tenderness. He denies any history of travel or contact with sick people. His laboratory results were remarkable for a total bilirubin of 4.7 mg/dL, direct bilirubin of 3.9 mg/dL, alkaline phosphatase of 583 U/L, and a lipase level of 56 U/L. A CT scan was performed showing pancreatic mass at the uncinate process with intrahepatic biliary dilatation with severe stenosis of the common hepatic duct. The gastroenterology service was consulted and requested abdominal MRI which confirmed the findings seen on the CT scan (Figure 1). 

**Figure 2:**
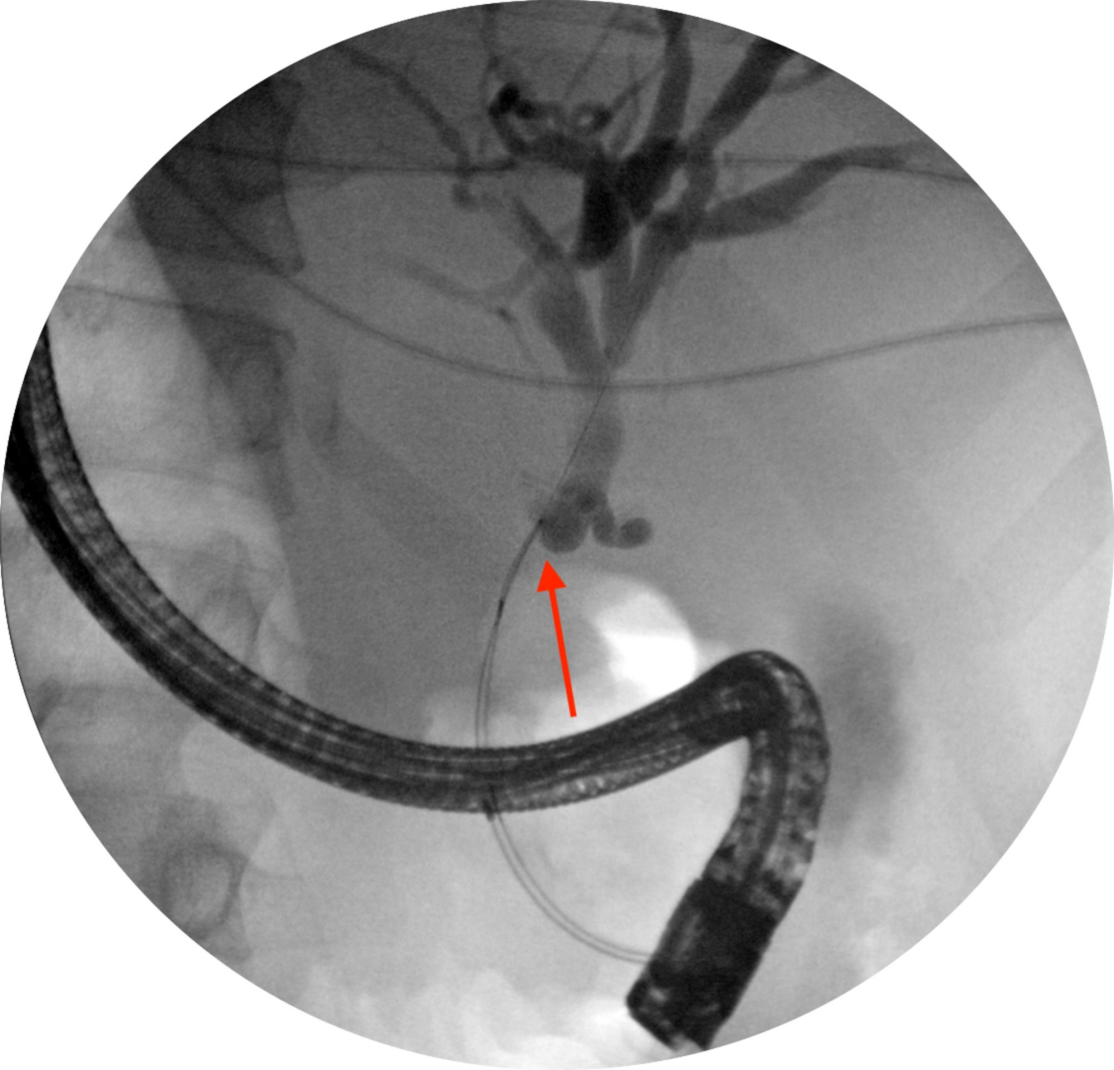
ERCP demonstrating 1.5 cm biliary narrowing in the middle of the common bile duct. ERCP, endoscopic retrograde cholangiopancreatography

Diagnostic and therapeutic endoscopic retrograde cholangiopancreatography (ERCP) was subsequently performed showing a 1.5 cm biliary narrowing in the middle of the common bile duct for which a 10 cm stent was placed (Figure 2). The narrowing was consistent with extrinsic compression by a lesion at the uncinate process of the pancreas. The surgical evaluation concluded that the lesion did not invade any vessels and that the patient was deemed appropriate for surgery. To complete the staging of the mass, endoscopic ultrasound (EUS) was performed.

**Figure 3:**
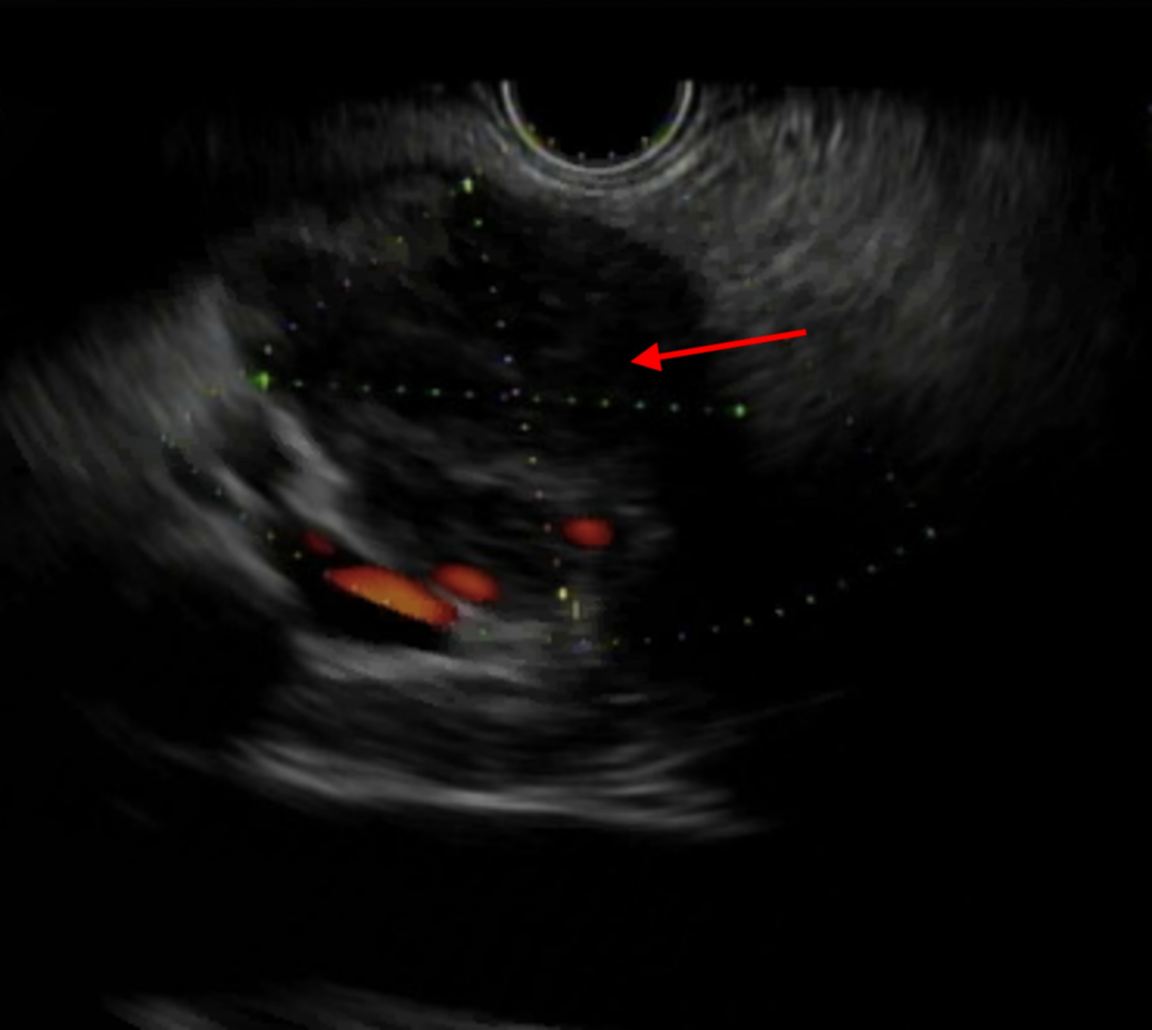
EUS demonstrating 30 mm x 26 mm hypoechoic lesion. EUS, endoscopic ultrasound

**Figure 4:**
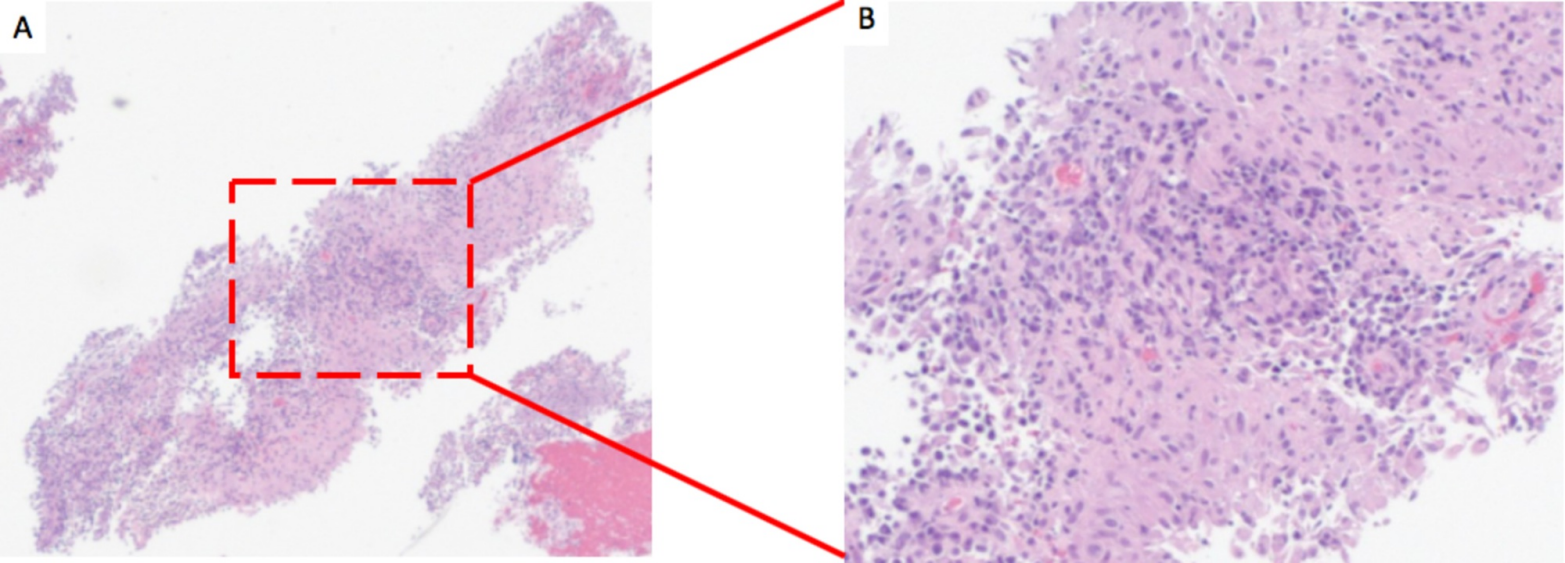
Pancreatic FNA showing abundant fibrinopurulent exudate and necrotizing suppurative granulomatous inflammation (A: H&E, 4X; B: H&E, 10X). FNA, fine needle aspiration

**Figure 5:**
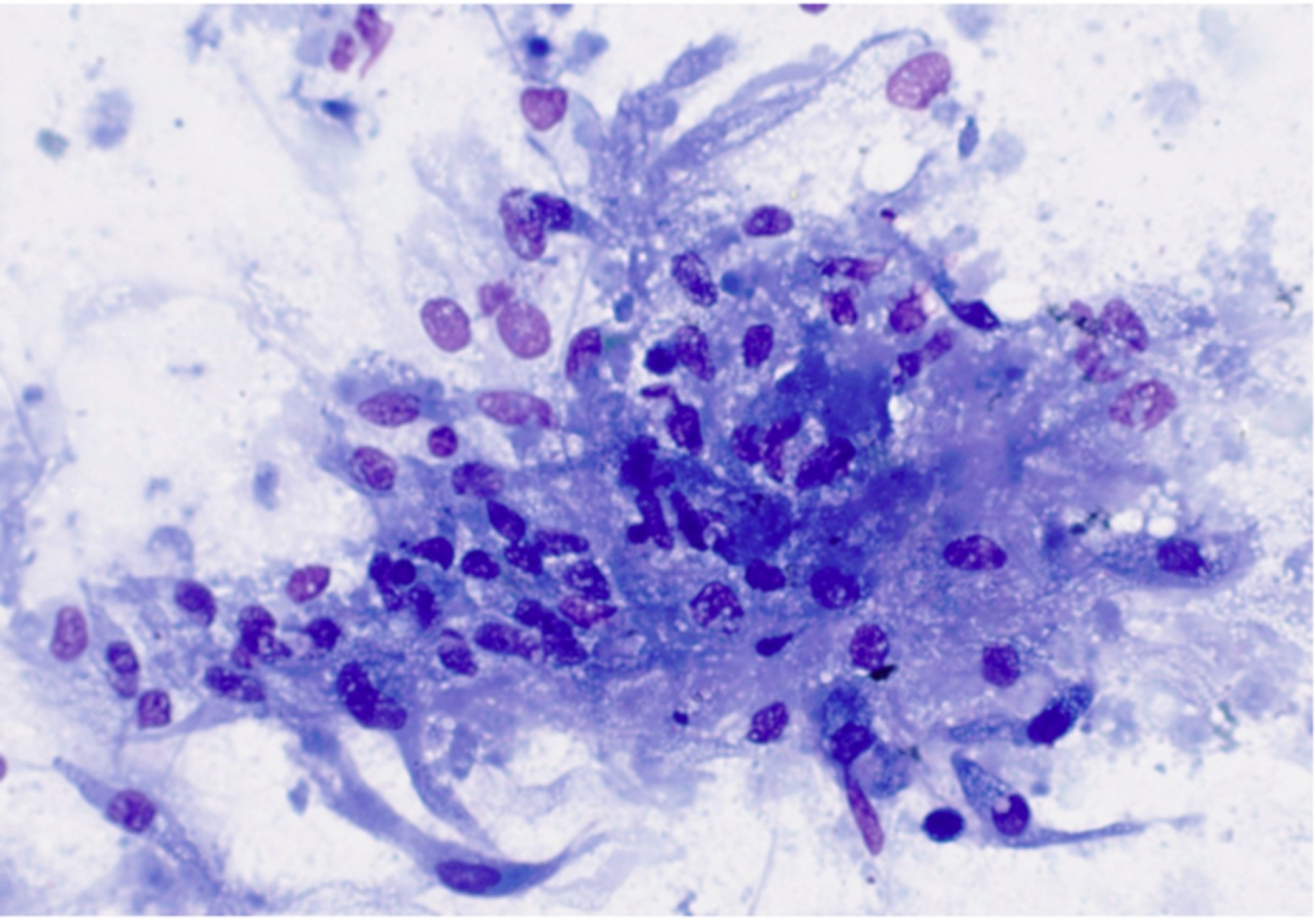
FNA of the pancreatic uncinate process showing necrotizing granulomatous inflammation (Papanicolaou stain, 40X). FNA, fine needle aspiration

The EUS-guided fine needle aspiration (FNA) of the mass revealed 30 mm by 26 mm hypoechoic lesion for which the pathology revealed the presence of necrotizing suppurative granulomatous inflammation (Figures 3-5). The stain of the pancreatic fluid was positive for a few acid-fast bacilli (AFB). The infectious disease (ID) team was consulted and they conducted a full infectious workup including HIV panel, hepatitis panel, blood cultures, sputum cultures, cytomegalovirus (CMV) deoxyribonucleic acid (DNA) quantitative polymerase chain reaction (PCR), Quantiferon-Tuberculosis gold test, Ova and parasites, histoplasma antigen, blastomyces serology, rapid plasma reagin (RPR) which all came back negative. All noninfectious causes of granulomatous disease such as antineutrophil cytoplasmic antibodies (ANCA) and angiotensin-converting enzyme (ACE) levels were negative. 

A CT thorax was ordered and showed hilar and mediastinal lymphadenopathy as well as a nodule in the right upper lobe centrally. The patient subsequently underwent a bronchoscopy with broncho-alveolar lavage and bronchial brushing which came back negative for *Pneumocystis jiroveci*, *Legionella pneumophilia*, *Mycoplasma pneumoniae*, fungal cultures, and AFB stain. Lymph nodes biopsies performed were only positive for extensive necrosis. Awaiting pancreatic fluid culture, a Mycobacterium PCR of the pancreatic fluid was analyzed and came back positive for *Mycobacterium tuberculosis* complex DNA. Three weeks later, cultures of the pancreatic fluid came back positive for *M. tuberculosis*. The patient was referred to the ID department for treatment and was scheduled for a follow-up with the gastroenterologist; however, the patient was lost to follow-up.

## Discussion

Foregoing the application of advent diagnostic modalities, pancreatic TB was an incidental finding during the evaluation of biopsy taken from patients undergoing surgical assessment for a presumed to be a pancreatic malignancy. Thus, a high index of suspicion is needed [5]. Our patient’s pancreatic TB was presumed to be a malignancy. Pancreatic adenocarcinoma is a devastating disease with a negative prognosis, even localized disease carries a 25%-30% survival rate [6].

The clinical approach in diagnosing pancreatic TB takes into consideration the compounds presenting symptoms and the relevant epidemiological factors (history of TB disease or exposure, living in endemic areas). Laboratory tests may show anemia, abnormalities in liver function test and bilirubin studies, and rarely diabetes mellitus [3, 7]. In this case, laboratory findings were suggestive of obstructive jaundice (elevated alkaline phosphatase, total and direct bilirubin). The definitive diagnosis, however, requires the demonstration of *M. tuberculosis* in the involved site via culture or nucleic acid amplification test (NAAT) [8].

The presence of extra-pancreatic TB may serve as a clue to pancreatic involvement. Up to 50% of patients with pancreatic TB were found to have abnormal chest radiographs, hence, chest radiographs are recommended [1]. The radiological test of choice is chest/abdomen CT as it allows for a comprehensive assessment of multiple organs simultaneously. Nevertheless, the characteristic radiographic findings in pancreatic TB may overlap significantly with findings suggestive of malignant etiology [1, 9]. 

The initial imaging modality is EUS which has easy feasibility and widely applied for the investigation of obstructive jaundice. A study conducted by Nagar reviewing 32 patients diagnosed with pancreatic TB showed that the most common findings are hypoechoic lesions (90%) in the body (56%) or the head (50%) of the pancreas. Peripancreatic lymph nodes involvement is fairly common occurring in 75% of the cases [7]. EUS is the preferred technique to obtain tissue biopsy which serves as a guide for performing FNA with a minor risk of causing needle tract spreading of tissue [10]. In our case, as the patient was deemed a candidate for surgery, EUS was only performed for the determination of resectability and staging. However, this approach carries a significant risk of misdiagnosis as it turned out that the result of the EUS-FNA changed the diagnostic as well as the therapeutic approach to our case.

Performing tissue biopsy for patients with intra-abdominal lymphadenopathy and pancreatic mass could be the only successful diagnostic modality. Positive PCR performed on the sampled tissue provides more diagnostic values (47%-96%) than AFB smear (0-62%) and AFB culture (19%-81%) [11]. 

The most frequent histopathological finding of specimens tested for pancreatic TB is necrotizing granuloma, which was found in this case [1, 5]. Although caseous necrosis is specific for TB, the detection of granuloma is not specific for pancreatic TB and all likelihood differential diagnosis should be examined. In this case, we investigated all infectious and noninfectious causes of granuloma and all tests were negative. 

The management of pancreatic TB is mainly a multidrug anti-TB therapy for between 6 and 12 months [12-13]. These patients still need to be followed up closely for subjective and objective response to therapy.

## Conclusions

Although pancreatic TB is a rare clinical entity, increased awareness of its existence in clinical conditions associated with immunocompetence is needed. Increased awareness among clinicians might dampen the healthcare cost associated with unnecessary diagnostic tests. We also encourage that EUS-guided FNA as a crucial step in the diagnostic algorithm in pancreatic lesions in order to spare patients from risky surgical procedures. 

## References

[1] Sharma V, Rana SS, Kumar A, Bhasin DK (2016). Pancreatic tuberculosis. J Gastroenterol Hepatol.

[2] Bhansali SK (1977). Abdominal tuberculosis. Experiences with 300 cases. Am J Gastroenterol.

[3] Auerbach O (1944). Acute generalized miliary tuberculosis. Am J Pathol.

[4] Paraf A, Ménager C, Texier J (1966). La tuberculose du pancréas et la tuberculose des ganglions de l'étage supérieur de l'abdomen [Tuberculosis of the pancreas and tuberculosis of the lymph nodes of the upper region of the abdomen]. Rev Med Chir Mal Foie.

[5] Chaudhary A, Negi SS, Sachdev AK, Gondal R (2002). Pancreatic tuberculosis: still a histopathological diagnosis. Dig Surg.

[6] Geer RJ, Brennan MF (1993). Prognostic indicators for survival after resection of pancreatic adenocarcinoma. Am J Surg.

[7] Nagar AM, Raut AA, Morani AC, Sanghvi DA, Desai CS, Thapar VB (2009). Pancreatic tuberculosis: a clinical and imaging review of 32 cases. J Comput Assist Tomogr.

[8] Aston NO (1997). Abdominal tuberculosis. World J Surg.

[9] Moujahid M, Ziadi T, Lamsiah T, Ouzzad O, Moudden A (2011). Tuberculose pancréatique: forme pseudotumorale À propos d'un cas [Pancreatic tuberculosis as a pseudotumor: a case report]. Sante.

[10] Song TJ, Lee SS, Park DH (2009). Yield of EUS-guided FNA on the diagnosis of pancreatic/peripancreatic tuberculosis. Gastrointest Endosc.

[11] Arai J, Kitamura K, Yamamiya A (2017). Peripancreatic tuberculous lymphadenitis diagnosed via endoscopic ultrasound-guided fine-needle aspiration and polymerase chain reaction. Intern Med.

[12] Ashok Kumar P, Singh G, Joseph JB, Swaminathan S, Venkatakrishnan L (2016). Pancreatic tuberculosis: a puzzle for physicians. A rare case and review of literature. J Clin Diagn Res.

[13] Pramesh CS, Heroor AA, Gupta SG, Krishnamurthy S, Shukla PJ, Jagannath P, Desouza LJ (2003). Pancreatic tuberculosis: an elusive diagnosis. HPB (Oxford).

